# Relationship between serum insulin and point-of-admission blood glucose of ill neonates at a tertiary health facility in Nigeria

**DOI:** 10.11604/pamj.2020.35.106.18929

**Published:** 2020-04-08

**Authors:** Emmanuel Oluwatosin Adeniji, Bankole Peter Kuti, Jerome Boluwaji Elusiyan

**Affiliations:** 1 Department of Paediatrics and Child Health, College of Health Sciences, Ladoke Akintola University of Technology, Osogbo, Nigeria; 2 Department of Paediatrics and Child Health, Obafemi Awolowo University, Ile-Ife, Nigeria

**Keywords:** Blood glucose, correlation, neonates, serum insulin

## Abstract

**Introduction:**

Optimal glucose metabolism is important in neonatal survival especially in the first days of life. Insulin play a significant role in maintaining blood glucose homeostasis. This study set out to determine the serum insulin levels of ill neonates as related to their point-of-admission blood glucose estimation at the Wesley Guild Hospital, Ilesa, Nigeria.

**Methods:**

Three hundred babies took part in the study. Blood glucose and serum insulin levels were assayed at admission using Accu-Chek Active glucometer^(R)^ and Accu-Ɔ-Bind ELISA Microwells^(R)^ respectively. Hyperglycaemia was defined as blood glucose ≥7mmol/L and hypoglycaemia as blood glucose <2.2mmol/L.

**Results:**

The median (IQR) age of the babies was 10.0 (0.5 - 70.0) hours with male to female ratio of 1.5:1. Seventy-four (24.7%) were preterms and 35 (11.7%) were small-for-gestational age. The mean (SD) blood glucose level of the babies was 4.1(2.1) mmol/L with a range of 0.6-13.4mmol/L. Hyperglycaemia and hypoglycaemia were observed in 18(6.0%) and 40(13.3%) babies respectively. The median (IQR) serum insulin level was 9.8(3.0-35.3) μIU/ml. There was weak positive correlation between serum insulin and blood glucose levels of the babies (r = 0.197, p = 0.001). Birth asphyxia was associated with lower serum insulin, while probable sepsis with relatively higher levels.

**Conclusion:**

Serum insulin level increases with increasing blood glucose in ill Nigerian babies at presentation to the hospital. Babies with asphyxia and sepsis particularly tend to have abnormal serum insulin at admission. Hyperinsulinaemia in ill babies may connote a compensatory mechanism to normalise abnormal blood glucose rather than playing significant role in its aetio-pathogenesis.

## Introduction

Blood glucose homeostasis is an important aspect of newborn physiology as abnormalities including hypoglycaemia and hyperglycaemia constitute significant risks to the newborn health [1, 2]. Insulin is secreted by the beta cells of the islet of Langerhans of the pancreas [3]. It causes rapid uptake, storage, and use of glucose by the tissues of the body especially the muscles, adipose tissue, and liver [4]. The counter-regulatory hormones are hormones that oppose the action of insulin leading to increased blood glucose levels [4]. They include glucagon, cortisol, growth hormone, adrenaline and noradrenaline. The levels of the counter-regulatory hormones are increased during stressful clinical conditions [3]. Neonatal hyperglycaemia (NNH) and its relationship with serum insulin in ill neonates is largely understudied especially in the West African sub-region. Many studies have attributed increased level of stress hormones which are known to stimulate gluconeogenesis as being important in the pathogenesis of hyperglycaemia in ill neonates [1, 5, 6] but the importance of serum insulin in this respect needs to be further studied. There is paucity of studies relating blood glucose levels in ill newborns and the respective serum insulin levels at the point of admission especially in Nigeria. Knowing the serum insulin levels in ill babies with hyperglycaemia may justify insulin use or otherwise early in the management of NNH. The aim of the study was to determine the point-of-admission serum insulin and its relationship with the blood glucose levels in neonates admitted to a Nigerian tertiary health facility.

## Methods

The study was a prospective cross-sectional study carried out at the Special Care Baby Unit (SCBU) of the Wesley Guild Hospital (WGH), Ilesa, Osun State, Southwest Nigeria. Ethical approval for this study was obtained from the Ethics and Research Committee of the Obafemi Awolowo University Teaching Hospital (OAUTH) with approval number ERC/2016/03/17. Babies aged 0-28 days admitted to the SCBU of the WGH irrespective of gestational age, birth weight and initial diagnosis were recruited into the study.

**Study location:** the WGH is one of the units of the OAUTH. The SCBU consists of eight glass-walled cubicles that can admit up to 32 babies. The ward is manned by six clinicians including a consultant paediatrician, 3 resident doctors and 2 house officers. These are assisted by 15 nurses and other supportive members of staff. Three hundred babies aged ≤28 days admitted to the SCBU of the WGH were recruited into the study at the point of admission into the SCBU. Exclusion criteria included babies who had major congenital anomalies or parental refusal of consent. History obtained about the recruited babies included the age at presentation, parent's socioeconomic class (using Oyedeji's classification) [7], place of antenatal care, place of delivery, mode of delivery, gestational age (GA) and the mother's parity. These were documented in the proforma designed for the study. Physical examination of each baby was done and significant signs were documented in the proforma.

The weight of the babies were classified into normal (2.50 - 3.99kg), low birth weight (LBW) (<2.50kg) and macrosomia (>05; 4.00kg) [8]. Babies were also classified as preterm (GA <37 weeks), term (GA between 37-42weeks), and postterm (GA >42 weeks). The babies were also classified into small-for-gestational age (SGA) i.e. weight <10^th^ percentile of the expected for the gestational age, appropriate-for-gestational age (AGA) when the weight is between the 10^th^ and 90^th^ percentile, and large-for-gestational age (LGA) when weight is >90^th^ percentile using an intrauterine growth chart [8]. Babies with sepsis were further classified as presumed sepsis, probable sepsis, and confirmed sepsis. Babies with confirmed sepsis not only had risk factors or clinical features of sepsis, but they also had bacteria growth on blood culture [9]. Presumed sepsis were babies with one or more risk factors to suggest sepsis including prolonged rupture of membrane, peripartum fever, or chorioamnionitis but yet to manifest clinical features of sepsis [9]. Babies with probable sepsis were babies with clinical features to suggest sepsis such as fever, hypothermia, poor suck, lethargy, respiratory distress etc. [9].

**Blood glucose and serum insulin determination:** peripheral venous blood was collected using aseptic procedure from each baby following venepuncture at the dorsum of the hand. A drop of venous blood was used for cot-side measurement of blood glucose using the Accu-Chek Active^®^ glucometer (Roche Diagnostics GmbH, Germany) while two millilitres of blood sample was collected in a plain bottle. Blood glucose value >6.9mmol/L was taken as hyperglycaemia while blood glucose value <2.2mmol/L was taken as hypoglycaemia [2, 5, 10, 11]. The two ml of blood sample that was collected in the plain bottle was centrifuged at 3000rpm for five minutes shortly after collection. The supernatant serum was then separated into another plain bottle and immediately stored inside the freezer in the hospital laboratory at temperature of -20° centigrade. The supernatant serum was then transported in ice packs to the Chemical Pathology Laboratory of the Obafemi Awolowo University Teaching Hospital, Ile-Ife where the serum insulin was determined by a Chemical Pathologist using the Accu-Ɔ-Bind Enzyme-Linked Immunosorbent Assay (ELISA) Microwells which was manufactured by Monobind Inc. Lake Forest, CA 92630, USA.

**Data analysis:** the data for the study was analyzed with Statistical Package for Social Sciences (SPSS) for windows version 23.0 (IBM Corp. Armonk 2015, NY, USA). Descriptive statistics including measures of central tendency like mean with standard deviation (SD) and median with interquartile range (IQR) were used to summarize normally and non-normally distributed continous variables respectively, while proportions and percentages were used to summarize categorical variables such as sex, and age range. Differences in serum insulin level of the various categories of babies which were not normally distributed were assessed using the Mann Whitney U test (U) or Kruskal Wallis test (k) as appropriate. Spearman's correlation was used to determine the relationship between serum insulin level and the blood glucose of the babies. Statistical significance was set at p value <0.05 at 95% confidence interval.

## Results

Over a six-month study period (January to June 2017), 300 babies were recruited into the study.

**Age of the babies at presentation:** the median (IQR) age of the babies at presentation was 10.0 (0.5-70.0) hours. Majority (59.0%) of the babies presented within 24 hours of delivery, while 37 (12.3%) presented after 7 days of life.

**Sex:** there were 179 (59.7%) males and 121 (40.3%) females among the study participants. The male to female ratio was 1.5: 1.

**Parental socio-economic class:** one hundred and fifty-eight (52.7%) of the recruited babies were born to parents from the middle socio-economic class while 111 (37.0%) babies were born to parents from upper class. Only 31 (10.3%) were from the low socio-economic class.

**Maternal age:** the mean (SD) maternal age was 29.4 (6.2) years with a range of 17 to 51 years. The highest proportion (82.4%) of the mothers were in the age range 20-35 years.

**Some obstetric characteristics and the babies' classification:** the obstetric characteristics of the mothers and the babies' classification are shown in Table 1.

**Table 1:** Obstetric characteristics and the babies’ classification

Variables	Frequency n = 300	Percentage (%)
Gestational Age		
Preterm	74	24.7
Term	218	72.6
Post term	8	2.7
Classification based on weight		
Normal	204	68.0
LBW	85	28.3
Macrosomia	11	3.7
Weight for Gestational Age		
AGA	254	84.6
SGA	35	11.7
LGA	11	3.7
Maternal Parity		
Primipara	120	40.0
Multipara	162	54.0
Grandmultipara	18	6.0
Booking Status		
Booked	123	41.0
Unbooked	177	59.0
Place of Delivery		
Inborn	146	48.7
Outborn	154	51.3
Mode of Birth		
Vaginal Delivery	183	61.0
Caesarian Section	117	39.0

**Gestational age (GA):** two hundred and eighteen (72.6%) of the babies were term while 74 (24.7%) were preterms. The mean (SD) GA of the babies was 37.7 (2.9) weeks with range of 26 to 44 weeks.

**Weight for gestational age:** two hundred and fifty-four (84.6%) of the babies were AGA, 35 (11.7%) were SGA and 11 (3.7%) were LGA.

**Maternal parity:** this ranged from one to seven with a mean (SD) of 2.2 (1.3). One hundred and twenty (40.0%) of the mothers were primiparous, while 162 (54.0%) were multiparous.

**Place of antenatal care:** the mothers of 123 (41.0%) babies received antenatal care (ANC) at the WGH (booked) while the mothers of the remaining 177 (59.0%) babies were unbooked at the WGH.

**Place of delivery:** one hundred and forty-six (48.7%) of the babies were delivered at the WGH (inborn) while the remaining 154 (51.3%) were outborns. Among the outborns, 58 (37.7%) were delivered at maternity centres, 47 (30.5%) were delivered at private hospitals, 25 (16.2%) were delivered at general hospitals while 15 (9.7%) and 9 (5.8%) were delivered at mission houses or at the mother's homes respectively.

**Mode of delivery:** one hundred and eighty-three (61.0%) of the babies were delivered by vaginal delivery while the remaining 117 (39.0%) were delivered through caesarian section.

**Gestational diabetes mellitus in mother:** five (1.7%) of the mothers had gestational diabetes mellitus (GDM).

**Serum insulin measurements:** the median (IQR) serum insulin for all the babies studied was 9.79 (3.0 -35.3) μIU/ml with a range of 0.1 to 383.5μIU/ml.

### Serum insulin levels in the different categories of babies

**Sex:** the median (IQR) serum insulin level for the male babies in this study was 10.7 (3.0 - 34.0) μIU/ml while that of female babies was 8.9 (3.2-38.0) μIU/ml. There was no statistically significant difference between the median (IQR) serum insulin levels in both groups of babies (Mann Whitney U = -0.069, p = 0.945).

**Age:** the babies that were older than or equal to 168 hours had the highest median (IQR) serum insulin level of 25.6 (7.3-43.3) μIU/ml. The median (IQR) serum insulin levels of the babies aged 72-167 hours, 24-71 hours, and babies less than 24 hours of age were 10.6 (3.3-47.2), 6.3 (2.0-34.0) and 6.8 (3.0-34.0) μIU/ml respectively. The age of the babies at presentation was significantly related with the median (IQR) serum insulin level (k = 8.602, p = 0.035).

**Gestational age:** the median (IQR) serum insulin of the post term babies was 34.0 (15.5-53.8) μIU/ml, while the median (IQR) serum insulin of term and preterm babies were 10.1 (3.1-35.2) and 6.4 (2.7-35.1) μIU/ml respectively. However, there was no significant difference in the median (IQR) insulin level in the various groups (k = 4.968, p = 0.083).

**Classification based on weight:** the median (IQR) serum insulin levels of the LBW babies was 5.0 (2.5-34.0) μIU/ml, that of normal birth weight was 10.9 (3.1-35.0) μIU/ml while that of the macrosomic babies was 5.2 (2.0-36.0) μIU/ml. There was no statistically significant difference in the median (IQR) serum insulin levels in the various birth weight categories (k = 3.991, p = 0.136).

**Weight for gestational age:** the median (IQR) serum insulin levels of the AGA babies was 10.0 (3.0-35.1) μIU/ml, median (IQR) serum insulin levels of SGA babies was 5.0 (3.0-35.0) μIU/ml, and median (IQR) serum insulin levels of LGA babies was 5.2 (2.0-36.0) μIU/ml. There was no statistically significant difference in the median (IQR) serum insulin levels in the different groups of babies (k = 0.426, p = 0.808).

**Serum insulin levels in the different categories of clinical diagnosis of the babies:** the median (IQR) serum insulin level in different clinical diagnosis was summarized in Table 2. The median (IQR) serum insulin level among babies with probable sepsis was 24.0 (7.3-43.2) μIU/ml while the median (IQR) serum insulin level among babies with confirmed sepsis was 9.9 (3.5-34.0) μIU/ml. The median (IQR) serum insulin level was significantly higher in babies with probable sepsis (k = 9.348, p = 0.025) compared with other groups of babies. The median (IQR) serum insulin level was significantly lower among babies with birth asphyxia compared with babies without birth asphyxia (U = -2.082, p = 0.037).

**Table 2:** Serum insulin level of babies with some clinical diagnosis

Variables (n)	Median (IQR) serum insulin in μIU/ml	Test statistics*	p-value
^#^Sepsis			
Probable sepsis (n=44)	24.0(7.3-43.2)	9.348**	0.025
Confirmed sepsis (n=15)	9.9(3.5-34.0)		
No sepsis (n=175)	6.8(2.8-35.0)		
Presumed sepsis (n=66)	6.4(3.0-34.0)		
Anaemia (n=63)	10.3(4.3-34.3)	-0.689	0.491
No anaemia (n=137)	8.4(3.0-36.0)		
Jaundice (n=41)	8.2(3.9-38.0)	-0.569	0.569
No jaundice (n=159)	9.9(3.0-35.3)		
Seizures (n=53)	7.9(4.3-41.2)	-0.861	0.389
No seizures (n=147)	9.9(3.0-35.0)		
Birth asphyxia (n=145)	6.2(3.0-31.0)	-2.082	0.037
No birth asphyxia (n=155)	12.6(3.8-40.1)		

* Mann-Whitney U test used

** Kruskal Wallis test

**Figure 1:**
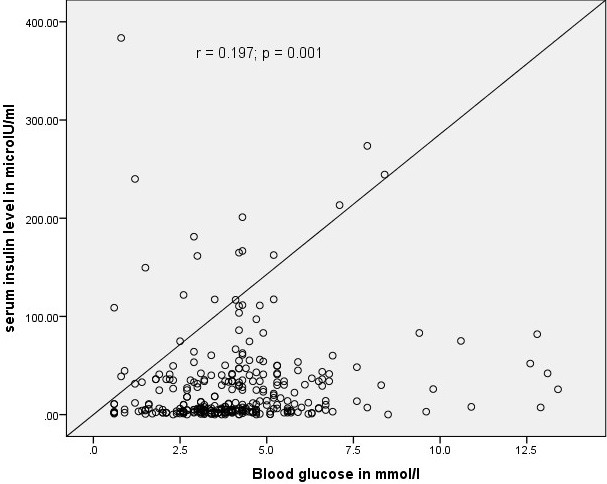
Scatter plot of the relationship between serum insulin level and blood glucose of the study participants

**Serum insulin measurements and relationship with blood glucose levels:** the median (IQR) serum insulin level for the hyperglycemic babies was the highest: 36.0 (7.7-82.2) μIU/ml. The least median (IQR) serum insulin level was found among the hypoglycaemic babies: 5.6(2.0-35.3) μIU/ml. This difference was statistically significant (k = 9.072, p = 0.011). The median serum insulin in the different blood glucose categories was summarized in Table 3. There was weak positive correlation between the random blood glucose and the serum insulin levels of the babies (r = 0.197, p = 0.001). Figure 1 shows a scatter plot of the relationship between the serum insulin levels and random blood glucose of the babies.

**Table 3:** Serum insulin measurements and relationship with blood glucose levels

Blood glucose categories	Median Serum insulin levels	Kruskal Wallis test	p value
Hyperglycaemia	36.0 (7.7-82.2) μIU/ml	9.072	0.011
Normoglycaemia	8.6 (3.0-34.0) μIU/ml		
Hypoglycaemia	5.6(2.0-35.3) μIU/ml		

## Discussion

The age of the babies in this study was significantly related to serum insulin levels as the babies that were older than seven days tend to have higher serum insulin levels. This is in agreement with the finding of Abdel-Azeem *et al.* [12] who also noted that serum insulin level tend to increase with the age of the babies. This is possibly because by seven days of life most mothers would have established breastfeeding. It is expected that with established or adequate feeding, the insulin secretion is increased to ensure adequate glucose uptake, use, and storage [3, 4]. The present study showed that serum insulin level in the babies with probable sepsis was significantly higher than that of the other groups of babies. To the best of the investigator's knowledge, no study was found to relate neonatal sepsis with elevated serum insulin. However, it is possible that since this study showed that probable sepsis was a risk factor for neonatal hyperglycaemia, serum insulin was most likely elevated as a compensatory mechanism to correct hyperglycaemia in those babies. In addition, serum insulin could possibly be elevated in babies with sepsis in reaction to the effect of stress and other counter-regulatory hormones like glucagon and cortisol [3-5].

These counter-regulatory hormones mobilise stored glucose from the liver and muscles which insulin tends to catabolise [3-5]. Could insulin be a biomarker of sepsis in newborn like C reactive protein and procalcitonin which are known biomarkers of sepsis [13]. This will be a good future research. Contrary to the investigator's expectation [14-16], there was no significant difference in the serum insulin levels of babies who were born low birth weight or macrosomic and those who had normal birth weight. Also, the serum insulin level was not significantly related to whether baby was small, appropriate or large for gestational age. These may be due to the small proportion of macrosomic, large for gestational age and small for gestational age babies compared to other groups of babies. Furthermore, in this study, the babies were recruited at different presenting ages when other factors such as inadequate lactation by mothers leading to reduced breastmilk intake must have set in which could have brought down the insulin level to normal, unlike the cord blood estimation of insulin level done in them at birth [14-16].

It was also shown in this study that the serum insulin level was not significantly related to the gestational age of the babies. This was against the finding of Wang *et al.* [17] in the United States of America who found that the serum insulin level was higher in preterm babies. The reason for this difference was because the babies recruited into the present study were recruited at different ages unlike the study by Wang *et al.* [17] in which babies were recruited at birth. Also, the proportion of preterm babies in this study was fewer compared to those of term babies. This study equally showed that serum insulin level was significantly lower in babies who had birth asphyxia. This was contrary to previous studies, most of which implicated birth asphyxia as a cause of high serum insulin levels [18-20]. This was possibly because a major portion of the recruited babies for this study were not admitted immediately after birth even when they had birth asphyxia, hence, the serum insulin most likely had reduced after an initial rise. The explanation for this is the negative feedback mechanism which is normally stimulated in the body to stop insulin secretion when it is elevated [3, 21]. Furthermore, when there is high level of insulin, it predisposes to hypoglycaemia which also turns off the insulin secretion thus reducing its level [3, 18].

The current study showed that babies with hyperglycaemia have significantly higher level of serum insulin more than the babies with normoglycaemia (36.0 versus 8.6μIU/ml). This was also supported by Verhoeven *et al.* [22] who also found elevated serum insulin in hyperglycaemic babies compared to normoglycaemic babies (30.8 versus 8.3μIU/ml). This shows that insulin does not constitute an important factor in the pathogenesis of hyperglycaemia at point of admission in most neonates, but it may be elevated as a compensatory mechanism to reduce the blood glucose back to normal [3]. Also, because there is elevated counter-regulatory hormones which is thought to be responsible for stress hyperglycaemia, there is associated relative insulin resistance [23].

## Conclusion

Serum insulin level increases with increasing blood glucose in ill Nigerian babies at presentation to the hospital. Babies with asphyxia and sepsis particularly tend to have abnormal serum insulin at admission. Hyperinsulinaemia in ill babies may connote a compensatory mechanism to normalise abnormal blood glucose rather than playing significant role in its aetio-pathogenesis.

### What is known about this topic

- The age of the babies was significantly related to serum insulin levels as the babies that were older than seven days tend to have higher serum insulin levels;
- The babies with hyperglycaemia had significantly higher level of serum insulin more than the babies with normoglycaemia;
- There was positive correlation between the blood glucose and serum insulin levels.

### What this study adds

- Serum insulin level in the babies with probable sepsis was significantly higher than that of the other groups of babies;
- This study equally showed that serum insulin level was significantly lower in babies who had birth asphyxia compared with babies without birth asphyxia;
- Serum insulin may play more of a compensatory role in reaction to high blood glucose levels.

## Competing interests

The authors declare no competing interests.
